# Biotoxin Detection Using Cell-Based Sensors

**DOI:** 10.3390/toxins5122366

**Published:** 2013-11-29

**Authors:** Pratik Banerjee, Spyridon Kintzios, Balabhaskar Prabhakarpandian

**Affiliations:** 1 Division of Epidemiology, Biostatistics, and Environmental Health, School of Public Health, The University of Memphis, 338 Robison Hall, 3825 Desoto Avenue, Memphis, TN 38152, USA; 2 School of Food Science, Biotechnology and Development, Faculty of Biotechnology, Agricultural University of Athens, Iera Odos 75, Athens 11855, Greece; E-Mail: skin@aua.gr; 3 Bioengineering Laboratory Core, Cellular and Biomolecular Engineering, CFD Research Corporation, 701 McMillian Way NW, Huntsville, AL 35806, USA; E-Mail: bxp@cfdrc.com

**Keywords:** biosensor, toxin, mycotoxins, marine toxins, botulinum toxins, cell-based sensors, cell-based assay, cytotoxicity

## Abstract

Cell-based biosensors (CBBs) utilize the principles of cell-based assays (CBAs) by employing living cells for detection of different analytes from environment, food, clinical, or other sources. For toxin detection, CBBs are emerging as unique alternatives to other analytical methods. The main advantage of using CBBs for probing biotoxins and toxic agents is that CBBs respond to the toxic exposures in the manner related to actual physiologic responses of the vulnerable subjects. The results obtained from CBBs are based on the toxin-cell interactions, and therefore, reveal functional information (such as mode of action, toxic potency, bioavailability, target tissue or organ, *etc.*) about the toxin. CBBs incorporate both prokaryotic (bacteria) and eukaryotic (yeast, invertebrate and vertebrate) cells. To create CBB devices, living cells are directly integrated onto the biosensor platform. The sensors report the cellular responses upon exposures to toxins and the resulting cellular signals are transduced by secondary transducers generating optical or electrical signals outputs followed by appropriate read-outs. Examples of the layout and operation of cellular biosensors for detection of selected biotoxins are summarized.

## 1. Introduction

Biosensors are devices that incorporate biological entities for analyses of target analytes [1]. The biological elements embedded in the sensor interact with the test substance and generate measurable signals detectable by a recorder [2,3,4,5]. Different types of biological entities have been used on sensor platforms, including (but not limited to), antibodies, nucleic acids, enzymes, protein ligands, molecular imprints, receptors, living cells of both prokaryotic and eukaryotic origins. Cell-based biosensors (CBBs) recruit viable whole cells as the sensing unit to report the presence of a particular analyte or a group of analytes. These sensors may use bacteria, yeast or higher eukaryotic cells including vertebrate or mammalian cells. CBBs utilize the principles of cell-based assays (CBAs) and integrate that to an enclosed device platform [2,4,5,6] (Table 1). Therefore, CBBs can be construed as the “on-site” application enabler of a CBA for analyte testing. These biosensors can record the deviation from normal cellular activities of the “sensing cells” as a result of exposure to an analyte, and this information can be used to identify the analyte. Since the premise of any CBB is critically dependent on the existence of a corresponding CBA, in this review we have, in many places, used these two terms interchangeably (as well as the assay results). However, we must recognize and underscore the necessity of cross-disciplinary collaborations between biologists, physicists, chemists, material scientists and engineers to translate the knowledge of CBAs for toxin detection to build automated and user-friendly CBB platforms.

The information about the *biological activity* of toxic substances including biotoxins can be obtained by cell-based sensors. These sensors, unlike other nucleic acid or antibody-based ones, respond to the toxins in a *physiologically relevant* manner, yielding information on the mechanism of action, and toxicological outcome of exposures. Also, CBAs provide much broader and complex functional information such as global information about gene expressions, protein synthesis, apoptotic or necrotic cell death compared to nucleic acid and immunochemical methods [2]. Information obtained by a CBA (and CBB) can provide insight into a mechanism of toxicant or pathogenic effect, which in turn facilitates not only detection but also agent classification. For example, toxins are classified based on their mode of action such as (i) membrane pore-forming toxins (hemolysin), diarrheagenic toxins (activating secondary messenger pathways—cholera toxin), superantigens (activating immune responses—staphylococcal enterotoxin B), neurotoxins (botulinum toxin) and protein synthesis inhibitory toxins (Shiga toxin). Any such toxins, when introduced to a CBB, would trigger an appropriate cellular response which is typical to the toxin’s (or similar class of toxins’) *mode of action*. The cellular response pattern from CBB can then be linked to the toxin (or toxins), thereby enabling subsequent detection of the same. This ability of CBBs to detect a broad range of analytes in a single assay to provide a “yes” or “no” response is particularly useful for the first line of screening. The inherent weakness of conventional electrical, chemical or biochemical sensors utilizing antibodies or DNAs/RNAs for categorical detection is that most of these sensors are designed to detect a specific agent. Therefore, a sensor specifically designed to detect the presence of agent A will not detect the presence of agent B. This chemical selectivity of the sensors is highly desirable when it comes to “identification” of the agents. However, sensors of this kind with a high degree of selectivity or discriminatory power suffer significant limitations when used as an “early warning” test method or a broad spectrum screening tool [7,8]. On the contrary, CBBs with the ability to “sense” a broad-range of analytes in a single assay have the ability to conform to challenges associated with emerging pathogens and unknown or little-known toxins which current detection methods (that depend on known chemical characteristics or molecular recognition of target organisms, toxins or substances) are unable to handle [3,4,6].

**Table 1:** Overview of the main components of representative CBBs used in toxicity assays.

Cell-Based Biosensors (CBBs)					
Microbial CBB			Invertebrate/Vertebrate/Mammalian CBB		
Sensor cell	Detection mode	Analyte [Reference]	Sensor cell	Detection mode	Analyte [Reference]
*E. coli* DH5α (pTOLGFP), (pTOLLUX)	Fluorescence, Bioluminescence	BTEX [9]	Hybridoma B-lymphocyte Ped-2E9	Fluorescence/colorimetry	Listeriolysin O (LLO), *B. cereus* crude toxin, cytolysin, α-hemolysin, phospholipase C [10,11]
*Pseudomonas putida* KT2440	Electrochemical	Aromatic hydrocarbons [12]	Green monkey kidney (Vero)	Bio-electric	Aflatoxin M1 [13]
*E. coli* DH5α (pJAMA-arsR)	Bioluminescence	Heavy metals (As, Sb) [14]	Hepatic (HepG2)	Optical/colorimetry	Marine toxins (azaspiracid-1, pectenotoxin-2, okadaic acid) [15,16]
*P. putida* JS444	Amperometry	Organophosphate nerve agents [17]	Neuronal (Neuro2a)	Optical/colorimetry	Marine toxins (azaspiracid-1, pectenotoxin-2, okadaic acid) [15,16]
*S. cerevisiae* Y190 (modified)	Amperometry	Endocrine disruptor compounds [18]	Lung fibroblast (V79)	Optical/colorimetry	Mycotoxin (14 different types) [19]
*E. coli* (modified)	Amperometry	Genotoxicity [20]	Neuronal (mouse embryonic frontal cortex and spinal cord tissues)	Electric/microelectrode array (MEA)	Botulinum neurotoxin (BoNT/A) [21]
*Salmonella typhimurium* (modified)	Amperometry	Genotoxicity [22]	Vero	Optical/colorimetry	Mycotoxin (T-2, ZEN) [23]
*Ralstonia eutropha* ENV307 (pUTK60)	Bioluminescence	PCBs [24]	Neuronal (PC12)	Fluorescence/FRET	BoNT/A and BoNT/E [25]

Historically, the concept of employing living cells for sensor applications started in the early to mid-1980s, the term “cell-based biosensor” was still in its infancy during this period, and mostly referred to sensors incorporating bacteria, yeasts or plant cells [26,27,28]. The sensing strategies were mostly electrochemical and more particularly amperometric [26]. Living microorganisms (bacteria or yeast) were used to interact with the test substances as a physiological sensor [29] or as biocatalysts for herbicide detection [28]. As compared to bacterial or plant cells, the applications of mammalian or higher eukaryotic cells as “sensors” were fewer. In one of those few reports during the 1980s, Slabbert *et al.* (published in 1984) reported that the oxygen uptake rates of the Buffalo green monkey kidney cell line can be used for rapid (10 min) detection of selected water toxicants [30]. The importance of this study was that mammalian cells were used as “sensors” and the authors presented their findings as a biosensing strategy rather than a cell-based assay. In their landmark study, Giaever and Keese (1984) demonstrated that the electrical impedance of mammalian fibroblast cells with their growth substrate (gold electrode) can change as a result of cellular movements [31]. During the early stage of development of CBBs, several research groups across the world conducted studies to understand the behavior of living cells under different toxicant and other analyte exposures to construct biosensing strategies [26,32]. Over the past three decades, living cell-based biosensing has emerged as an extremely powerful tool for toxicity analyses, and there are several cell-based sensors commercially available [3]. 

The present review summarizes the major research efforts on the development of detection methodologies for different toxins utilizing CBBs based on different CBA principles as the scientific and physiological basis of the sensor. This review also sheds light on the advantages, challenges, and the future of cell-based sensors in toxin detection applications.

## 2. Choice of Biological Cells for Toxin Testing

Choosing the proper cell type is the single most important consideration for cell-based biosensing. The choice of sensing cells is dependent on the chemical and biological nature of the toxic substances to be tested. Both prokaryotic (bacteria) and eukaryotic (yeast, invertebrate and vertebrate) cells have been used to create CBBs (Table 1). Based on the different cellular characteristics a diverse group of toxic agents can be screened by CBBs utilizing either prokaryotic or eukaryotic cells. Regardless of the cell types used, the underlying principle of cell-based sensing of toxins or toxic substances is always to reveal the extent of toxicity of the test substance on the living system [3,33]. Even though most of the biotoxins are detected using higher eukaryotic cells (such as insect, fish, mammalian or other vertebrate origins), bacterial cells are widely used worldwide to detect organic and inorganic environmental pollutants including genotoxic, ecotoxic, endocrine disrupting, xenobiotic compounds, to name a few [34]. Keeping in mind the important role that microbial CBBs played during the last fifty years, this review will briefly discuss the key aspects of microbial CBBs in toxicant detection.

### 2.1. Microbial CBBs

**Figure 1:**
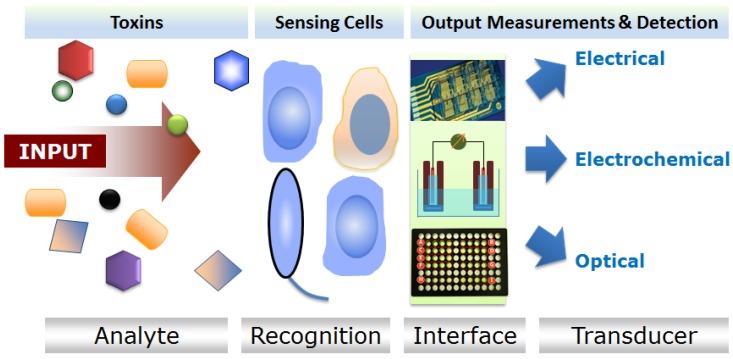
Generalized working principle of cellular sensors.

Live microorganisms respond to various chemical toxicants that are present in an environment, hence sensors utilizing these types of cells (microbial cell sensors) have been commonly used in environmental monitoring [33]. Engineered bacterial cells were successfully employed to detect chemicals in different samples since the early 1990s [35] to present dates [36,37]. Most of these engineered bacterial cell sensors are optically active (luminescent or fluorescent) and report the presence of toxicants by changes in light emissions [34]. In one type of bacterial CBB, inducible luciferase genes (bacteria *luxCDABE* or firefly *luc*) are expressed in the sensing cells. This type of bacterial CBB is considered to be “lights-on” as a result of exposure to a specific analyte [33,34]. In another type, cells are either natural carriers of luminescence or fluorescence genes or proteins or transformed to express these genes constitutively. The basal level of luminescence or fluorescence decreases (“lights-off”) in these CBBs upon toxic exposures [34]. The frequently used commercial ecotoxicity sensor, “*Microtox*” utilizing *Aliivibrio fischeri* (formerly known as *Vibrio fischeri*), is such an example of a “lights-off” bacterial CBB [38]. Another class of microbial CBB generates measurable electrochemical signals when challenged with specific toxicants [39]. The major modes of signal transduction and detection mechanisms of the electrochemical microbial sensors include amperometric, potentiometric, or conductometric mechanisms [39] (Figure 1). It is to be noted that most of the toxic analytes detected by microbial cell sensors can be classified under the broad categories of metal (including heavy metals) [40,41,42], naphthalene and salicylate [43,44,45], BTEX compounds (benzene, toluene, ethylbenzene, and xylenes) [9,46,47], polychlorinated biphenyl (PCB) [48], phenols [49,50], surfactants [51], aromatic hydrocarbons [12,52], genotoxic compounds [22,53,54], to name a few. These types of sensors are employed mostly in ecotoxicological studies involving water and environmental monitoring. 

### 2.2. Higher Eukaryotic (Invertebrate and Vertebrate) CBBs

Cell-based sensors utilizing cells of mammalian or other multi-cellular higher eukaryotic (invertebrate and vertebrate) origins constitute another major category of toxicity sensor. This class of CBBs is amenable to a wide range of *biotoxin* detection [3]. Both wild-type or engineered cells are used to construct the sensing element of a higher eukaryotic CBB [2]. For wild-type cells, the sensing mechanisms may include toxin-induced cell membrane damage and cytotoxicity [2,55], receptor ligand interactions [3], *etc.* Like microbial CBBs, higher eukaryotic CBBs may also harbor genetically modified cells that are inducible by toxins [56]. These cells can also be engineered to carry analyte-specific receptors on the membrane (“membrane engineered”) to interrogate specific target toxins [13]. The signal transduction mechanisms for this type of cell-based sensors have some similarity with the microbial cell-sensors. Like microbial CBBs, eukaryotic or mammalian CBBs may utilize optical (luminescence or fluorescence) signal reading to evaluate toxigenic exposures [10,56]. Several excitable mammalian cells (of neuronal or cardiac origin) used on micro-electrode array-integrated CBB platform provide electrical readouts to deduce toxicity results [57,58,59]. Many vertebrate and invertebrate cells used on CBBs for toxin detection measure electrical impedance [60,61], electrophysiology [62], or electric potentials [13,63] of cell and neighboring culture medium or matrices. It is important to note that the higher eukaryotic cell-based sensors can detect several biological toxins that microbial CBBs are unable to detect. One such example is toxins of microbial origin [10].

Microbial CBBs are usually easier to handle as compared to mammalian and vertebrate/invertebrate CBBs. Also, bacterial cells are more robust and remain viable in harsher environments. Because of these advantages, bacterial or microbial CBBs are frequently used in ecotoxicity measurements and environmental toxicity evaluations. On the other hand, higher eukaryotic CBBs offer a closer physiological relevance to human or animals, and they elicit detectable toxicity to toxins of animal, microbe, and plant origins. Because of these factors, eukaryotic CBBs remain the choice of biotoxin detection, despite the difficulty of growing these cells outside of specialized laboratories. 

## 3. CBBs for Biotoxin Detection: Examples from Major Application Areas

In this review, a synopsis of some recent examples of biotoxin detection using CBB (or CBA) is provided with particular focus on the foodborne toxins (bacterial or fungal); toxins related to marine environment (mostly algal); and the botulinum neurotoxins. For a more detailed discussion on cellular biosensors for diagnostics of different toxic agents (not limited to biotoxins) and pathogens, the readers are encouraged to consult comprehensive reviews published earlier [2,3,4,5,6,34,37,39,64].

### 3.1. Detection of Foodborne Toxins

#### 3.1.1. Foodborne Bacterial Toxin Detection

**Figure 2:**
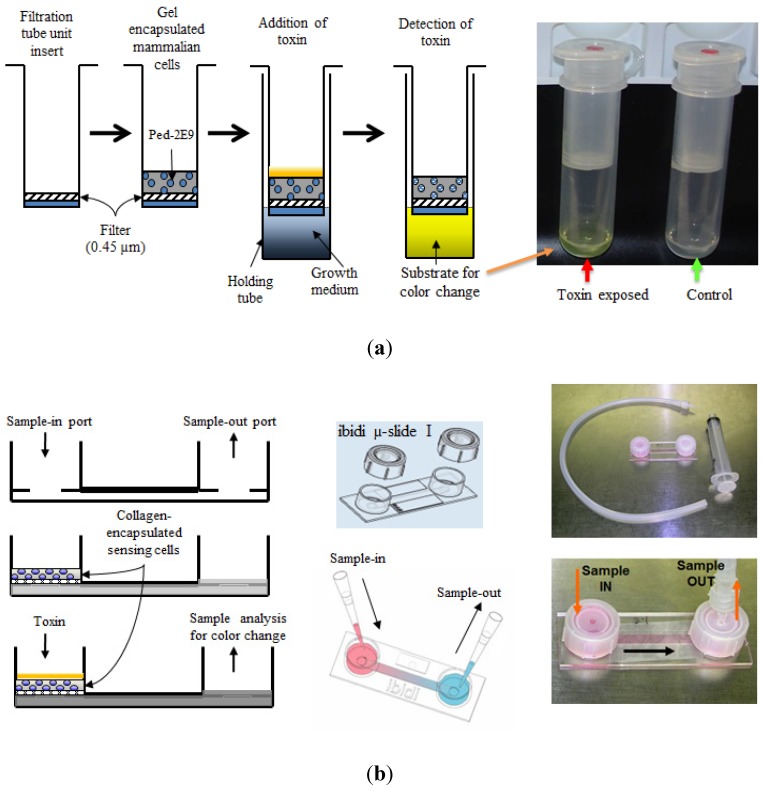
Schematic design of CBB prototypes used for detection several hemolytic/cytolytic toxins. (**a**) A centrifugal filtration tube unit-based biosensor device and the sequence of operations; (**b**) A hand-held µ-Slide device prototype. Samples containing different toxins were introduced into the sample chambers followed by detection using a colorimetric alkaline phosphatase enzyme assay. Adopted with some modifications from [10].

Since the 1990s, CBBs are used frequently in detection of toxins from foodborne pathogens [3,65]. Bhunia and co-workers reported a novel murine hybridoma cell-based assay for hemolytic and membrane damaging toxin listeriolysin O (LLO) from the leading foodborne pathogen *Listeria monocytogenes* [11,55,65,66]. Based on these initial studies involving a toxin-induced cytotoxicity, later a CBB platform was developed which utilized the ability of *Listeria* to infect and produce cytotoxicity to murine hybridoma B cells (Ped-2E9) which can be detected by the release of alkaline phosphatase (ALP) [10,67]. Employing this hybridoma B-cells, Banerjee and co-workers developed three different biosensor prototypes consisting of three-dimensionally encapsulated sensing cells [10,11] (Figure 2 depicts two of the three prototypes). Several cytolytic or hemolytic toxins (e.g., crude toxin preparations from pathogenic *L. monocytogenes* Scott A and *B. cereus* A926, α-hemolysin from *Staphylococcus aureus*, phospholipase C from *Clostridium perfringens* and cytolysin from *Stoichactis helianthus*) were detected in different food products spiked with different concentrations of these toxins with a limit of detection (LOD) of 10 ng/gm of food [10]. Conversely, the cholera toxin from *Vibrio cholerae* lacking cytolytic properties (no hemolysis in blood agar plate) induced only minimal cytotoxicity indicating the ability of this sensor to respond to only one category (*i.e*., cytolytic or hemolytic) of toxin (categorical detection). Moreover, this type of CBBs provided important functional information about the toxins rather than just detecting them. When exposed to LLO, this CBB revealed a higher rate of apoptosis in sensor Ped-2E9 cells. Another interesting finding was that the sensor demonstrated a higher necrotic cell death than apoptotic cell death with all other cytolytic toxins tested [10]. These results from CBB were in agreement with previous reports that cytolytic toxins caused necrotic fate of the mammalian cells by inducing rapid membrane damage [11,66,68]; the same mechanism was believed to be responsible for the overwhelming necrotic death of Ped-2E9 cells on CBB platform when exposed to cytolysins. It is also important to note that the emetic toxin producing *B.cereus* F4810 [69] shows lower hybridoma cytotoxicity as compared to diarrheagenic strains (*B. cereus* strains A926, MS1-9, ATCC-14579) [70,71,72]. This may indicate the fact that tripartite hemolysin BL (HBL) toxin from *B. cereus* diarrheagenic strains have a more profound membrane-damaging activity on the sensing Ped-2E9 cells utilized in the CBB compared to emetic toxin (cereulide)-producing strains [73]. 

#### 3.1.2. Mycotoxin Detection

**Figure 3:**
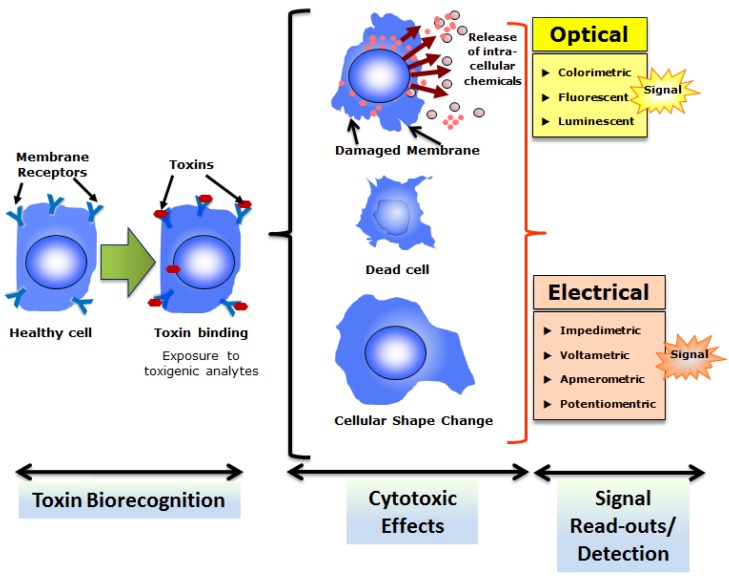
Overall strategy of cell-based biosensing exploiting cytotoxicity of select toxins. Cells with appropriate recognition elements (often membrane receptors) respond to respective toxins which result in cytotoxicity.

CBAs are routine procedures for screening or toxicological analyses of different mycotoxins. Most of these CBAs rely on the ability of mycotoxins to induce cytotoxicity in mammalian or higher eukaryotic (invertebrate and vertebrate) cells. It must be noted that many cellular sensors are developed based on the cytotoxic properties of different bacterial toxins, mycotoxins, or other type of toxins (such as algal toxins discussed in the next section). The general scheme of CBBs based on cytotoxicity is depicted in Figure 3. In a CBB platform, the cytotoxic effects may release extracellular chemicals (such as enzymes), cause cell death (necrosis or apoptosis), or engender changes in cellular morphology (such as shape change) that can be detected as estimators of cytotoxicity (and toxins) by appropriate optical or electrical transducers. A list of different types of cells utilized in the cytotoxicity assay for mycotoxins (from *Fusarium* spp.) can be found in a comprehensive review published earlier [74]. It is also important to note that application of multiple cell lines of different origins to test same mycotoxins reveals more accurate estimations of toxicity particularly for toxins with moderate to low cytotoxic potency [74,75]. Recently a study reported the generation of reactive oxygen species (ROS) and mammalian heat shock protein (Hsp) 70 expression in green monkey kidney (Vero) cells upon concurrent exposures to *Fusarium* mycotoxins, ZEN and T-2 toxin as a detection strategy to evaluate toxicity of these mycotoxins [23]. Vero cells were exposed to varying concentrations from 0 to 100 nM ZEN and T-2 toxins individually and in combinations for 24 h followed by evaluation of cytotoxicity by a standard 3-4,5-dimethylthiazol-2-yl,2,5diphenyltetrazolium bromide (MTT) test. The results revealed a synergistic nature of cytotoxicity indicating possible health risk with elevated toxicological potency of these two interactive toxins of fusarial origin. In another recent study, the cytotoxic potency of fourteen (14) structurally different mycotoxins were tested using a hamster lung fibroblast cell line (V79) [19]. This study demonstrated the feasibility of using lung cells to assess the locally occurring inhalation-related toxicity caused by mycotoxins in the lung. A CBB was recently developed based on the well-characterized Bioelectric Recognition Assay (BERA) for ultra-sensitive and instantaneous detection of aflatoxin M1 (AFM1) [13]. BERA is a novel CBB that detects electric signals from membrane engineered whole cells (mammalian or higher eukaryotic) suspended in a gel matrix in response of target analytes [76,77]. The BERA-based cellular sensor demonstrated the ability to detect AFM1 at concentrations as low as 5 pg/mL (5 ppt) within 180 seconds [13]. 

### 3.2. Detection of Marine Toxins

A number of mammalian or other higher eukaryotic (invertebrate and vertebrate) cells show significant cytotoxicity when exposed several marine toxins. Therefore cytotoxicity-based detection assays have been utilized for the detection of many of these marine toxins such as paralytic shellfish poisoning (PSP) toxins including saxitoxin and derivatives, diarrheic shellfish poisoning (DSP) toxins including okadaic acid and dinophysis toxins, brevetoxin, ciguatoxin, palytoxin, pectonotoxins, and tetrodotoxin [78]. Complementary CBAs were developed for positive mouse bioassay showing atypical shellfish toxicity. These CBAs were found to be extremely useful to detect the causative toxins. In a recently conducted multi-laboratories collaborative study, three lipophilic marine toxins azaspiracid-1 (AZA1), okadaic acid (OA), and pectenotoxin-2 (PTX2) were exposed to cells derived from their target organs (intestinal Caco2, hepatic HepG2, and neuronal Neuro2a) [15,16]. The results of the study revealed that HepG2 and Neuro2a cells were responsive to AZA1. Also, the study revealed that shellfish (oyster or mussel) extracts spiked with OA exhibited the highest toxic effect on Caco2 cells (mean IC_50_ value ~ 1.5 mg DG/mL, which is equivalent to 27 nM OA); by using all the above cell-lines, the results from all collaborative labs were statistically significant for detection of shellfish extracts spiked with PTX2 [16]. It is important note that this study underscored the importance of CBA as a powerful assay method for atypical marine toxicity events and as a screening tool for selecting toxic chromatographic fractions for further detection and identification process [16]. A spinal cord neuronal network biosensor (NNB) cultured over a microelectrode array (MEA) was used successfully to detect brevetoxin-3 (PbTx-3) and saxitoxin (STX) from sea water matrix [79]. This NNB is special class of CBB incorporating neuronal cells. In a typical NNB, glial support cells and randomly seeded neurons are co-cultured on MEA substrate, which upon exposure to stimulants (such as toxins) results in spontaneous bioelectrical activity that includes neuronal spiking which can be recorded by electrophysiological recorders [79]. 

### 3.3. Detection of Botulinum Neurotoxins

**Figure 4:**
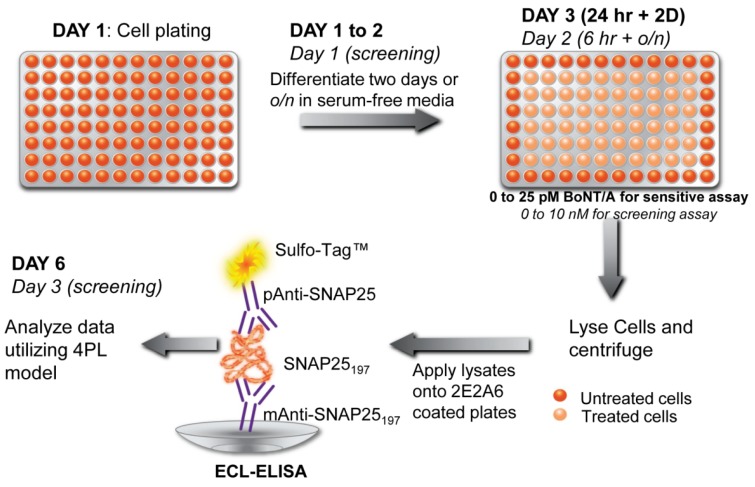
Detection scheme of botulinum neurotoxins A (BoNT/A) using a CBB coupled with a sandwich ELISA. In this cell-based potency assay, a sensitive and rapid screening (in italics) of BoNT/A was achieved using differentiated human neuroblastoma SiMa cells. Reproduced from [85] under the terms of the Creative Commons Attribution License.

Application of different cell-based assays and CBBs for detection of other major toxins including botulinum neurotoxins (BoNTs) has been extensively reported in scientific publications. For the BoNTs, CBA and CBBs are emerging as a powerful alternative to *in vivo* mouse lethality assay or *in vitro* ELISA, endopeptidase assays, or immuno-PCR methods [80]. CBAs eliminate several shortcomings of traditional BoNT assay such as the inability of ELISA to differentiate between active and inactive toxins or holotoxin and reduced toxin. Components of BoNT sample buffer components or human serum albumin (routinely used as pharmaceutical excipient preparations) interfere with endopeptidase assays causing false positive results [81]. The trend of CBAs in BoNT detection can be found in recently published comprehensive review articles by Pellett [82] and Dorner *et al.* [83]. Whitemarsh *et al.* [84] have pioneered the use of human-induced pluripotent stem cells (hiPSC) for BoNT detection, achieving the quantitative determination of different serotypes. In another recent report, a cellular sensor was described that utilized Förster Resonance Energy Transfer (FRET) methods to detect BoNT/A and BoNT/E [25]. In this FRET-based cellular sensor, transplantable rat pheochromocytoma PC12 cells were transfected with FRET-based substrates as fusions of green fluorescence protein (GFP) and *Discosoma* sp. red fluorescent protein (DsRED) containing full-length neuronal soluble N-ethylmaleimide sensitive factor attachment protein receptor (SNARE) protein SNAP-25. The genetically engineered sensors cells were treated with 1–10 nM BoNT/A for 72–96 hours and BoNT/E for 72 hours followed by acquisition of the FRET signals by a multimode plate reader. It should be mentioned, however, that PC12 is not optimal for a BoNT assay, not least due to its considerably low detection sensitivity compared to other available CBAs. Recently, a novel cell-based potency assay (CBPA) coupled with sandwich ELISA for BoNT/A detection was reported [85]. In this assay, differentiated human neuroblastoma SiMa cells were exposed to BoNT/A complex (with varying concentrations of 0.005 to 300 pM) for 24 h followed by a two-day incubation which resulted in the accumulation of SNARE protein SNAP25_197_. The accumulated SNAP25_197_ protein was detected by a sandwich ELISA assay with a monoclonal capture and polyclonal detection antibodies on a high-throughput electrochemiluminescence (ECL) detection platform (Figure 4). In another report, the application of cultured cortical neural networks on MEA as a biosensor for BoNT/A complex was demonstrated [86]. The results from this study showed that within 24 h after the toxin exposures there was an increase in the number of spikes per burst from the neural networks biosensor. However, considering the signal-nullifying effect of BoNTs on MEA-attached cells (by blocking neurotransmitter release), the practical usefulness of this approach is limited.

### 3.4. Other Applications

Cancer drug (chemotherapy) toxicity is a major issue and a very promising area for the application of CBBs and CBAs. Emerging technologies could be incorporated in current preclinical drug screening platforms in a rapid and cost-efficient manner never realized by the state of the art. *In vivo* pharmacological studies using animals provide information on complex intercellular and organ-to-organ effects. However, *in vivo* studies have many and significant disadvantages: they are expensive, labor intensive and time consuming, while ethical concerns have decreased their popularity in recent years. Existing CBA-based principles for toxicity screening can satisfy the basic requirements for assessing the potential side effects of a chemotherapeutical protocol, either by providing information on the number of total cells or the number of viable cells after administration of the toxicant or through the indirect measurement of cell viability (e.g., by measuring respirational activity or cell motility). A recent example of the potential application of CBAs in this field is given by Gopal *et al.* [87]. The authors have used an *in vitro* model of cortical networks (CNs), enriched in auditory cortex cells; to quantify cisplatin neurotoxicity and the protective effects of D-Met. Cisplatin at 0.10–0.25 mM induced up to a 200% increase in spontaneous spiking activity, while concentrations at or above 0.5 mM caused irreversible loss of neuronal activity, accompanied by cell death. Pretreatment with D-Met, at a concentration of 1.0 mM, prevented the cisplatin-induced excitation at 0.10–0.25 mM, caused sustained excitation without occurrence of cell death at 0.5 mM, and delayed cell death at 0.75 mM cisplatin.

## 4. The Challenges and Future of CBBs in Toxin Detection

The cell-based sensing of toxins offers several advantages that the other detection methods fail to fulfill. Despite the advantages of both CBAs and CBBs, there are several challenges that this screening strategy faces which can be found in detail in our previously published, comprehensive review articles on CBBs [2,3,5]. Here, we discuss some of the key limitations; firstly, these sensors are *not specific* as different toxins may evoke the same or a similar cellular response which the secondary transducers may not be able to differentiate. Therefore, when the exact identification of the toxin is a requirement, CBBs may not be the appropriate choice of detection method. To address the specificity issue, engineered sensing cells were developed, such as those in BERA sensors [4,13,76,77] whereby analyte-specific antibodies are inserted on the cell-membrane or in CANARY^®^ sensors [56,88] where specific antibodies are expressed on the cell surface by stable transfection. Another major issue with CBBs is related to cellular viability (especially for mammalian or vertebrate CBBs) for the practical field applications. The shelf life of live whole cells on a sensor platform is very limited, and prolonged storage under cryogenic conditions often reduce the viability as well as efficacy of sensing cells [2]. Mammalian or other multi-cellular higher eukaryotic (invertebrate and vertebrate) cells grown even in standard cell culture conditions require precise control of the microenvironment and are susceptible to minute changes in that environment. Thus, when used on a CBB platform, a rigorous standardization must be performed for these types of cells to acquire reliable results [3]. One of the most critical requirements for the wide use of CBBs for toxin detection is to increase the “working life” of the sensor cells outside of the standard laboratory conditions [2,3,5,55,65,89]. To maintain the viability of sensing cells on CBBs for an extended period, several measures have been taken by different research groups. For example, an approach of employing cells of non-mammalian origin was recently proposed [90]. In this study, Rainbow trout gill epithelial cells (RTgill-W1) were used for detection of toxic chemicals in drinking water. This cell type remained viable on the sensor biochip for 78 weeks at 6 °C in ambient air compositions. In another recent report [91], mammalian sensor cells were transfected to overexpress genes from an extremophile organism: this modification resulted in a 3.5-fold increase in viability of the engineered sensor cells at ambient conditions. It is obvious that the ability to keep sensors cells (invertebrate and vertebrate) viable for a prolonged period of time in an ambient environment is a critical need (as well a scientific challenge) for portable and field-ready CBB systems. Microfluidics based solutions or Lab-on-a-Chip (LoC) systems can readily address these issues. They require minimal volumes of samples and reagents, are fabrication friendly, cost effective, disposable and can be readily adapted for the common end point assays using optical or electrical detection [92,93]. In addition, their miniature sizes make them highly portable [94]. They can also be used for rapid screening of multiple toxins in a high throughput manner [92,95]. Finally, use of co-culture systems enable toxin detection in physiological relevant environment of the cellular architecture *in vivo* [96]. It is interesting to note that unlike higher eukaryotic cells, microbial cells (particularly bacterial) are more stable and resilient, and can withstand extreme conditions more so than their higher eukaryotic counterparts. Therefore, microbial cells could serve as alternatives in some toxin detections to fragile higher eukaryotic CBBs [2,3,64,65]. 

More significantly, however, CBAs and CBBs offer some unique advantages which other systems of toxin detection are unable to provide, prominently among them being the ability to sense the toxin according to their biological (hence physiological) functions. Moreover, a CBB can distinguish between active *versus* inactive toxins, can report bioavailability of toxicants, and can provide important information on the toxicological effects of the analytes. Since the signal(s) from CBBs is resultant of toxin–cell interactions, consequently unknown or emerging toxins with toxic effects can be screened using this system. Therefore, CBBs are perhaps the only *in vitro* testing methods that can detect unknown toxins [7]. With the ever-increasing demand to create a broad-based sensor (“universal sensor”) applicable to biosecurity, environment monitoring, or food and agricultural diagnostics, CBBs are thought to be the next major sensing mechanisms [3,97]. Since the translation of the basic understandings of CBAs must be “re-formatted” in a field-portable, automated, and user-friendly CBB platform, a close collaboration between an inter-disciplinary research approach involving biologists, chemists, physicists and engineers is therefore essential for the fruition of field-deployable CBBs with greater ability to sense toxins in physiological or sub-physiological concentrations from different matrices.
